# miR-146a suppresses cellular immune response during Japanese encephalitis virus JaOArS982 strain infection in human microglial cells

**DOI:** 10.1186/s12974-015-0249-0

**Published:** 2015-02-18

**Authors:** Nikhil Sharma, Ruhi Verma, Kanhaiya Lal Kumawat, Anirban Basu, Sunit K Singh

**Affiliations:** Laboratory of Neurovirology and Inflammation Biology, CSIR-Centre for Cellular and Molecular Biology (CCMB), Uppal Road, 500007 Hyderabad, AP India; Current Affiliation: Laboratory of Human Molecular Virology and Immunology, Molecular Biology Unit, Faculty of Medicine, Institute of Medical Sciences (IMS), Banaras Hindu University (BHU), 221005 Varanasi, India; National Brain Research Centre, Haryana-122050 Manesar, Haryana India

**Keywords:** JE, miR-146a, Neuropathogenesis, STAT1, NF-κB activity, ISG, Viral immune evasion

## Abstract

**Background:**

Japanese encephalitis virus (JEV) is the causative agent of Japanese encephalitis which is more prevalent in South and Southeast Asia. JEV is a neurotropic virus which infiltrates into the brain through vascular endothelial cells. JEV infects neurons and microglial cells which causes neuronal damage and inflammation. However, JEV also evades the cellular immune response to survive in host cells. Viruses are known to modulate the expression of microRNAs, which in turn modulate cellular immune response by targeting expression of antiviral genes. The aim of this study is to understand the anti-inflammatory role of miR-146a during JEV infection, which facilitates immune evasion.

**Methods:**

Human brain microglial cells (CHME3) were infected by JEV: JaOArS982 and P20778 strain, and expression of miR-146a were analyzed. Overexpression and knockdown studies of miR-146a were done to see the effect on NF-κB pathway and antiviral Jak-STAT pathway. Regulatory role of miR-146a on expression of interferon-stimulated genes was determined by real-time PCR and luciferase assays.

**Results:**

JEV infection elevated the expression of miR-146a in JaOArS982 strain which caused downregulation of TRAF6, IRAK1, IRAK2, and STAT1 genes. Exogenous overexpression of miR-146a led to suppression of NF-κB activation and abrogation of Jak-STAT pathway upon JEV infection which led to downregulation of interferon-stimulated genes (IFIT-1 and IFIT-2) and facilitated viral replication. JEV infection initially upregulated cytokine production and activated STAT1 activity but STAT1 levels reduced at later time point, which led to the downregulation of interferon-stimulated genes.

**Conclusion:**

Upregulation of miR-146a by JEV JaOArS982 strain leads to suppression of NF-κB activity and disruption of antiviral Jak-STAT signaling which helps the virus to evade the cellular immune response. This effect of JEV infection on miR-146a expression was found to be strain specific.

**Electronic supplementary material:**

The online version of this article (doi:10.1186/s12974-015-0249-0) contains supplementary material, which is available to authorized users.

## Introduction

Japanese encephalitis virus (JEV) is a mosquito-borne neurotropic virus which belongs to the family flaviviridae. JEV is mostly prevalent in South and Southeast Asia. JEV leads to death of 25% of infected patients whereas 50% of survivors suffer from neuronal damage, loss of memory, and cognitive dysfunction [1]. JEV causes encephalitis and death in patients. A comparative study between the different parts of the brain depicted that JEV has more affinity to the midbrain and thalamus [2]. JEV maintains a zoonotic life cycle where pigs are major reservoir hosts and mosquitoes act as vectors [3]. JEV infects macrophages and PBMCs [4] and infected macrophages help the virus to cross blood brain barrier [5]. However JEV also persists latently in T-lymphocytes [6] and PBMCs [7]. JEV has been also reported to suppress dendritic cell maturation and causes expansion of Treg cells [8]. Hence, JEV evades the cellular innate response which facilitates its survival in the host. JEV infects microglial cells and causes microglial activation which leads to neuronal damage due to inflammation [9]. Microglial cells are resident macrophages in the brain which harbor JEV. A study on mouse microglial cells demonstrated persistence of JEV in microglial cells and suggested that these cells may serve as reservoirs for virus [10].

MicroRNAs are the small regulatory RNAs which are 19 to 24 nucleotides in length and are reported to regulate the expression of around 60% of human genes [11]. MicroRNAs (miRNAs) lead to regulation of gene expression by binding to complementary sites present in 3′UTR of target gene via their seed region [12,13]. Viruses modulate the expression of cellular microRNAs [14] which may help the virus to augment its replication [15] or in evasion of cellular immune response [16]. Recently, the expression of miR-29b [17] and miR-155 [18] has been reported to be modulated upon JEV infection. Viruses like DENV, CHIKV and VSV have been reported to overexpress miR-146a which helps the virus to shut down inflammatory responses of cell [19,20,21]. miR-146a is a well-known anti-inflammatory microRNA which targets TNF receptor-associated factor 6 (TRAF6) and IL-1 receptor associated kinase-1 (IRAK1) and IRAK 2 genes [20]. These genes encode for various adaptor proteins involved in NF-κB activation. miR-146a has been also reported to target STAT1 gene which acts as a transcription factor for expression of interferon-stimulated genes [22]. miR-146a is induced by NF-κB activation, where induction of miR-146a targets genes involved in NF-κB activation and forms a regulatory negative feedback loop in monocytes [23]. miR-146a was found to be overexpressed in Tregs and its deficiency led to disruption of immunological tolerance in mice [24]. miR-146a overexpression leads to suppression of cellular inflammatory response and decrease in cytokine secretion [25,26].

In our study, we found that JEV-induced miR-146a upregulation led to the downregulation of TRAF6 and IRAK1 and IRAK2 genes and suppression of NF-κB activation along with decreased expression of pro-inflammatory cytokines. The initial activation of NF-κB by virus resulted in increased expression of miR-146a, which targeted the adapter molecules involved in NF-κB activation through a negative feedback loop. JEV-mediated miR-146a upregulation downregulated the STAT1 expression and abrogated the Jak-STAT pathway, which led to the decreased expression of interferon-stimulated genes (ISGs). This observation suggested the exploitation of cellular miR-146a by JEV to suppress cellular inflammatory responses in order to create favorable cellular environment for their survival.

## Materials and methods

### Cell culture

Human microglial cell line CHME3 was obtained as a gift from Dr. Anirban Basu (National Brain Research Centre, Manesar, Haryana). CHME3 cells were grown in Complete Dulbecco Modified Eagle Medium (DMEM) (#12100-046, Gibco, Rockville, MD, USA) with 10% heat-inactivated fetal bovine serum (16000–044; Gibco BRL) and 100 U penicillin and 100 μg/ml streptomycin (#10378016; Gibco-BRL). Porcine stable kidney cells (PS cells) for JEV Plaque Assay and C6/36 cells for JEV propagation were also cultured in Complete Dulbecco Modified Eagle Medium.

### JEV propagation and infection

JEV strains (JaOArS982 and P20778 Vellore strain) were given as a gift by Dr. Anirban Basu, NBRC which was further propagated in mosquito cell line C6/36 (*Aedes albopictus*). The 2 × 10^5^ cells were seeded in 75-cm^2^ flask and infected with JEV at MOI 0.1 in incomplete DMEM (without FBS and antibiotic) medium. The incomplete DMEM media was replaced by complete DMEM media 3 h post infection, and cells were incubated for 8 days in humidified 5% CO_2_ incubator at 28°C. The supernatant was collected, and the virus was precipitated using PEG virus precipitation kit (#ab102538; Abcam, Cambridge, MA, USA). Virus titer was determined by using plaque assay. For plaque assay, 2 × 10^5^ PS cells were seeded in six-well plates and different dilutions of virus (10^−3^ to 10^−9^) were used for infection. Three hours post infection, cells were washed with PBS and agarose overlay medium (2X incomplete DMEM, 5% FBS, 2% low melting agarose, and 1% penicillin-streptomycin) was added on cells and kept at 37°C incubator for 72 h. Later, the cells were fixed by 10% formaldehyde and the overlay was removed. The cells were stained with crystal violet stain, and plaques were counted to determine the virus titer. For infection experiments, 5 × 10^5^ CHME3 cells were seeded in 25 cm^2^ flask and infected by JEV at MOI 5 in incomplete DMEM. Three hours post infection, the media was replaced by complete DMEM and cells were harvested at 24 h post infection.

### miR-146a overexpression

CHME3 cells were seeded in six-well dishes, and 100 pmol of miR-146a seed sequence mimic (Bioserve, Hyderabad, India) was transfected by using Lipofectamine 2000 (#11668-019; Invitrogen, Carlsbad, CA, USA) according to manufacturer’s protocol. Scrambled seed sequence of miR-146a and mock Lipofectamine treatment were used as control. The sequence of miR-146a oligo and scramble has been mentioned in Table 1. The overexpression of miR-146a was confirmed by real-time PCR using TaqMan probe. The cells were harvested after 48 h post transfection for RNA isolation and Western blotting.

**Table 1:** **Sequence of RNA oligos used**

**Name of oligos**	**Sequences**
miR-146a mimic	UGAGAACUGAAUUCCAUGGGUU
Scramble	GGAUGUAUGCUGCUGCUAAUAA

### Anti-miR-146a (miR inhibitor) overexpression

CHME3 cells were transfected with 100 pmol of anti-miR-146a (#AM 10722, Ambion) along with 100 pmol Cy3-labeled scrambled anti-miR (#AM17011; Ambion) as negative control by using Lipofectamine transfection reagent. Cy3-labeled control enables to determine transfection efficiency into cells. Knockdown of miR-146a was confirmed by real-time PCR using Taqman probe. Cells were harvested 48 h post transfection.

### RNA isolation and real-time PCR

Qiagen miRNeasy kit (#217004; Qiagen, Venlo, Netherlands) was used for miRNA isolation from harvested cells. Complementary DNA (cDNA) synthesis was done by using multiscribe TaqMan reverse transcriptase (#4366596; Applied Biosystems, Waltham, MA, USA) with miR-146a specific primers. Real-time analysis of miR-146a level was done by real-time PCR (ABI VII A7 RT-PCR) by using miR-146a-specific TaqMan probe and universal PCR master mix (#4324018; Applied Biosystems). The expression of miR-146a was normalized by endogenous control RNU24 expression.

For estimation of IL-6, IFIT-1, and IFIT-2 transcript levels and viral RNA, total RNA was extracted by using RNeasy mini kit (Qiagen, Cat No. 74106) according to manufacturer’s protocol. Quantification of RNA was done, and cDNA was prepared by using superscript II (Invitrogen, Cat No. 11904-018) according to manufacturer’s protocol. List of primers used in this study is given in Table 2.

**Table 2:** **List of primers used for real-time PCR**

**Genes**	**Primer sequences**
IL-6	Forward: 5′ ACTCACCTCTTCAGAACGAATTG 3′
	Reverse: 5′CCATCTTTGGAAGGTTCAGGTTG 3′
TNF-α	Forward: 5′ CCTCTCTAATCAGCCCTCTG 3′
	Reverse: 5′GAGGACCTGGGAGTAGATGAG 3′
JEV NS3	Forward: 5′ AGAGCGGGGAAAAAGGTCAT 3′
	Reverse: 5′ TTTCACGCTCTTTCTACAGT 3′
GAPDH	Forward: 5′ ATGGGGGAAGGTGAAGGTCG 3′
	Reverse: 5′ GGGGTCATTGATGGCAACAATA 3′
IFIT-1	Forward: 5′ AGAAGCAGGCAATCACAGAAAA 3′
	Reverse: 5′ CTGAAACCGACCATAGTGGAAAT 3′
IFIT-2	Forward: 5′ CACATGGGCCGACTCTCAG 3′
	Reverse: 5′ CCACACTTTAACCGTGTCCAC 3′

### Western blotting

The harvested cell pellet was lysed in RIPA buffer (150 mM NaCl, 50 mM Tris-HCl, pH 7.5, 1% NP-40, 0.5% sodium deoxycholate, 0.1% SDS) with 1 μM PMSF and 1X proteCEASE-50 (#427P; G-Biosciences, St. Louis, MO, USA). The lysate was sonicated, and protein was quantified by bicinchoninic acid (BCA) assay. Equal amount of protein was loaded into each well, resolved on 12% SDS-PAGE gel, and transferred on PVDF membrane. Five percent skimmed milk in 1X TBS-Tween 20 was used for blocking the membrane. The membranes were then incubated in primary antibody (1:1,000) overnight followed by three washes with TBST each for 15 min. Later, the membrane was incubated in HRP-conjugated secondary antibody for 1 h and then washed thrice with 1X TBST (15 min each) and developed by using super-signal developing reagent as HRP substrate. Primary antibodies against TRAF6 (#8028; Cell Signaling Technology, Danver, MA, USA), anti-IRAK1 (#4504; Cell Signaling Technology), anti-IRAK2 (#4367; Cell Signaling Technology), anti-phospho-NFKB p65 (#3037; Cell Signaling Technology), anti-NFKB p65 (#4764; Cell Signaling Technology), anti-STAT1 (#9172 Cell Signaling Technology), anti-phospho-STAT1 (#8826 Cell Signaling Technology), anti-JEV NS1 (#ab41651; Abcam) and anti-β-tubulin (#ab6046; Abcam) antibodies were diluted in 5% BSA in TBST buffer. Goat-raised HRP-conjugated anti-rabbit secondary antibody (ab6721-1; Abcam) was used in 1:50,000 dilution.

### Luciferase assays

CHME3 cells were seeded in six-well dishes and were transfected with NF-κB luciferase reporter plasmid (1 μg) along with β-galactosidase (700 ng) vector by using Lipofectamine 2000. For miR-146a overexpression and anti-miR experiments, 100 pmol of scrambled miR and miR-146a and 100 pmol of Cy3-labeled scrambled anti-miR and anti-miR-146a were co-transfected along with plasmids, and luciferase activity was measured 48 h post transfection. For infection, cells were counted 24 h post transfection and infected with JEV (MOI 5). Cells were incubated for 24 h after infection and were lysed to measure luciferase activity. ISRE and IFN-β luciferase assay was also done in similar manner. Luciferase activity was measured 24 h post infection. For measuring the luminescence activity, cells were lysed in 1X lysis buffer provided by Luciferase Assay kit (#E4030; Promega, Madison, WI, USA) and luminescence was measured by adding luciferase assay reagent as per manufacturer’s protocol. Luciferase activity was measured in Perkin Elmer multiplate reader (Enspire 2300 Multimode plate reader). The luciferase activity was normalized by β-galactosidase activity. β-galactosidase activity was measured by using β-Galactosidase kit (#E2000; Promega, Madison, WI, USA) as per manufacturer’s protocol.

### ELISA of TNF-α

CHME3 cells were seeded into six-well plate, and 100 pmol of miR-146a was transfected by using Lipofectamine 2000. Scramble sequence was used as control. Twenty four hours post transfection, JEV infection was given, the supernatant was collected after 24 h, and ELISA was performed to determine secreted TNF-α level by using human TNF-α ELISA kit (#-KHC3011, Invitrogen) according to manufacturer’s protocol. For anti-miR experiments, 100 pmol of anti-miR-146a was transfected along with Cy3-labeled negative control prior to JEV infection.

### Statistical analysis

All experiments were done in triplicates, and comparison was made between all data sets by a one-tailed, unpaired Student’s *t*-test or one-way ANOVA. Data was considered significant when *P* < 0.05. * denotes *P* < 0.05, ** denotes *P* < 0.005, and *** denotes *P* < 0.001.

## Result

### miR-146a gets upregulated during JEV infection

**Figure 1:**
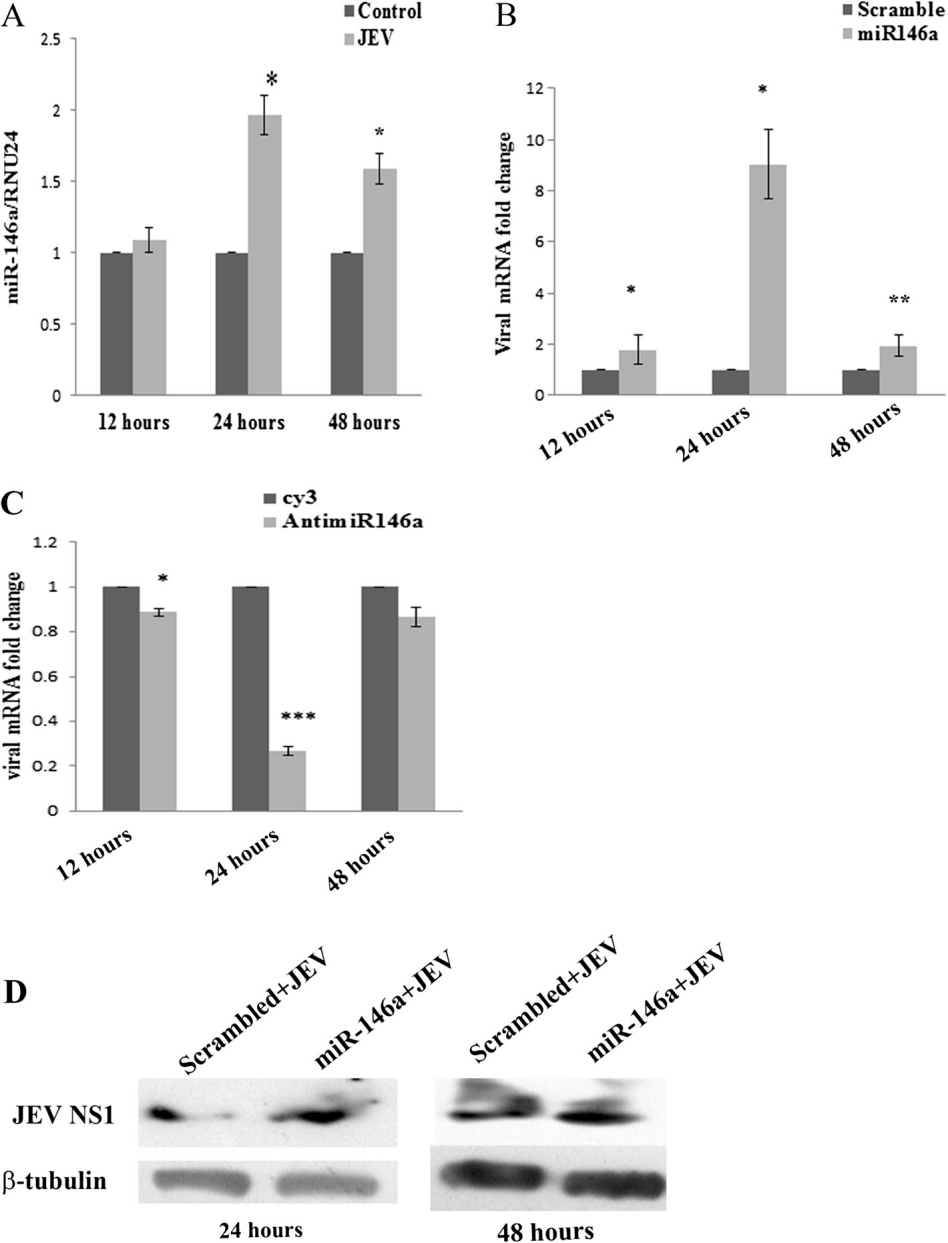
**JEV upregulates miR-146a, and miR-146a enhances viral replication.** JEV infection upregulates miR-146a in CHME3 cells. **(A)** Human brain microglial cell line CHME3 was infected with JEV (MOI-5), and cells were harvested at 12, 24, and 48 h post infection. qRT-PCR with Taqman probes were used to determine miR-146a levels. RNU24 was used to normalize the fold change in miR-146a levels as compared to uninfected control. **(B,C)** CHME3 cells were transfected with scramble sequence or miR-146a mimic sequence **(B)**, Cy3-labeled scrambled anti-miR or anti-miR-146a **(C)** and infected by JEV 24 h post transfection. The cells were harvested at 12, 24, and 48 h post infection for RNA isolation. Viral RNA level was determined by RT-PCR using JEV NS3 specific primers. The fold change was normalized by GAPDH RNA levels. Fold change was determined by 2^−∆∆C^_T_ method. For statistical analysis, scrambled miR and Cy3-labeled scrambled anti-miR group was used as control. **(D)** Western blots showing upregulation of viral NS1 protein in miR-146a overexpressing cells. A 100 pmol of scrambled sequence and miR-146a mimic was used. Scramble + JEV group was used as control for comparison. The cells were infected by JEV (MOI-5) and harvested 24 and 48 h post infection. All the experiments were done three times independently. The data are shown as mean ± SE from three independent experiments. The fold change is statistically significant. The fold change is significant where *denotes *P* < 0.05, **denotes *P* < 0.005, and ***denotes *P* < 0.001.

Many groups have reported increment of miR-146a levels after viral infection [19,20]. The levels of miR-146a were checked after JEV infection (JaOArS982 strain) (MOI 5) in CHME3 cells. TaqMan microRNA assay was used to quantify miR-146a levels, and 1.9-fold increase was observed in miR-146a levels, 24 h post JEV JaOArS982 strain infection (Figure 1A). The expression levels of miR-146a were determined at two time points of 12 and 24 h post infection but found no change in miR-146a levels at 12-h time point. In order to study the strain specificity, we also checked the miR-146a levels in cells infected by P20778, Vellore strain of JEV. As reported by Pareek *et al.* [27], the reduced expression of miR-146a was observed in CHME3 cells infected by P20778, Vellore strain (Additional file 1: Figure S1A). Thus, we conclude that endogenous miR-146a is not relevant for replication of P20778 strain as it downregulates miR-146a post viral infection. Our findings supported the strain-specific effect of JEV on the expression of miR-146a in CHME3 cells. miR-146a is a well-known anti-inflammatory molecule, which suppresses the release of pro-inflammatory cytokines in activated microglial cells [28,29]. Therefore, we checked the downstream effects of miR-146a upregulation upon JEV infection in human microglial cells and its effect on JEV replication.

### miR-146a enhances JEV replication

Overexpression of miR-146a creates anti-inflammatory milieu in the cell; therefore, we were interested to determine its effect on viral replication in cells. We overexpressed 100 pmol of miR-146a and scramble mimic and gave JEV infection (JaOArS982 strain) after 24 h. The viral RNA level at three time points (12, 24, and 48 h) was checked. We found significant increase by ninefold in viral RNA levels at 24 and a very marginal increase at 12 and 48 h post infection when compared to scramble control (Figure 1B). The effect of miR-146a on viral replication was seen to be time point dependent and got neutralized at 48 h after infection. To further ensure that miR-146a enhances replication of JaOArS982 strain, we suppressed endogenous miR-146a by transfecting anti-miR-146a and gave JEV infection to cells. We found a decrease in viral copy number at all three time points (12, 24, and 48 h) but a significant dip of 75% was observed at 24 h post infection (Figure 1C). Additionally, the effect of miR-146a was checked on expression of viral proteins. We observed the induced expression of the JEV non-structural protein 1 (NS1) in miR-146a overexpressing CHME3 cells as compared to scramble transfected cells 24 h post infection (Figure 1D). Induced expression of NS1 levels was observed at 48 h post infection, which may be due to accumulation of viral proteins (Figure 1D). We also determined the effect of miR-146a on P20778 Vellore strain and found enhanced levels of viral RNA by RT-PCR in miR-146a overexpressing cells (Additional file 1: Figure S1B). These enhanced levels of P20778 viral RNA was also due to anti-inflammatory environment created by miR-146a overexpression. Hence, miR-146a creates a virus-friendly milieu in cells, which promotes JEV replication.

### JEV infection downregulates TRAF6, IRAK1, and IRAK2 genes

**Figure 2:**
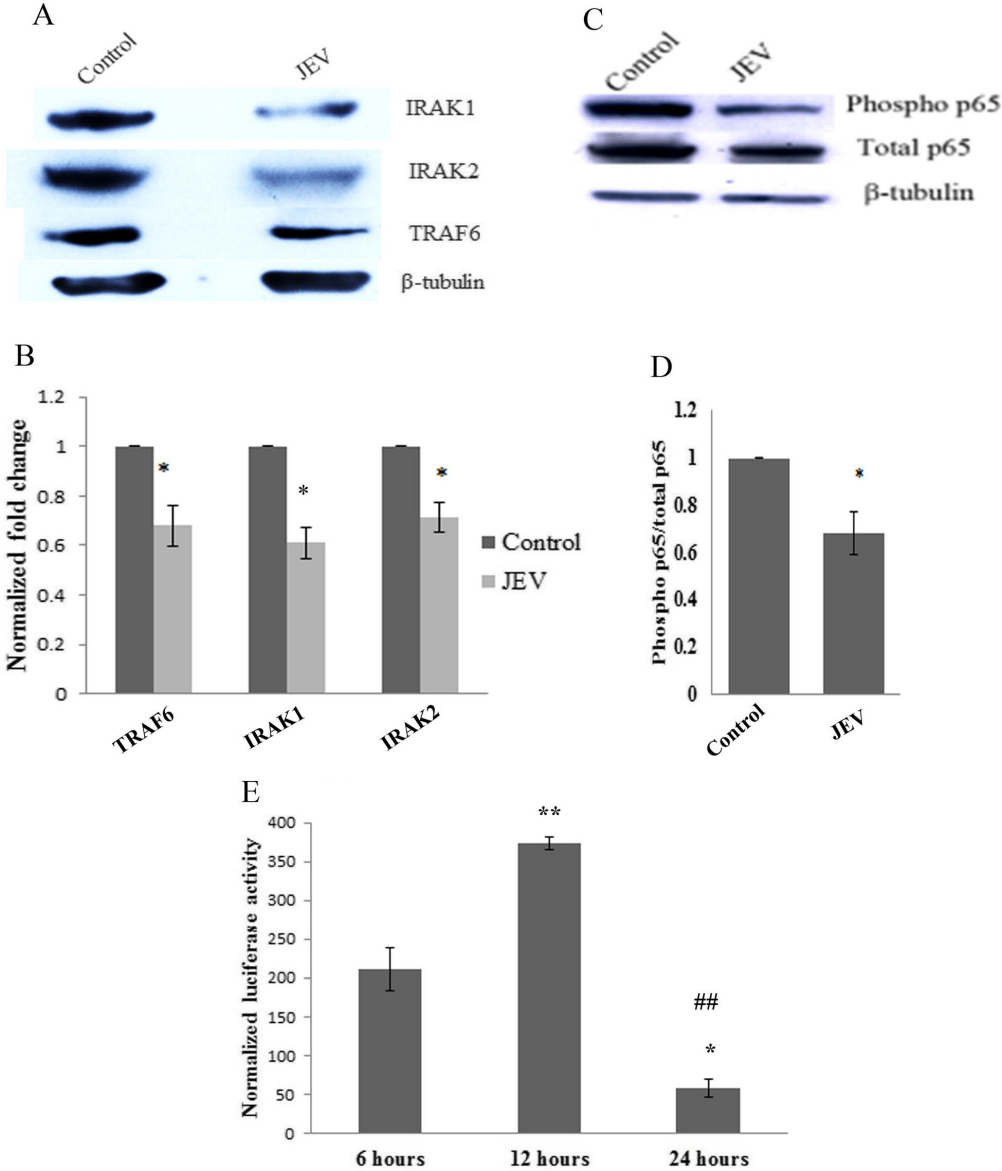
**JEV downregulates TRAF6, IRAK1, IRAK2, and NF-κB activation.** CHME3 cells were infected by JEV (MOI-5) and harvested 24 h post infection. **(A)** Western blots showing downregulation of TRAF6, IRAK1, and IRAK2 genes. Uninfected cells were used as control. **(B)** Graph bars representing densitometry plot depicting downregulation of TRAF6, IRAK1, and IRAK2 genes post JEV infection. The image density of blots was normalized by β-tubulin by using ImageJ software. **(C)** Western blot showing downregulation of phospho-p65 upon JEV infection. **(D)** Densitometry plot showing decreased phosphorylation of p65 subunit of NF-κB. The ratio of phospho/total p-65 decreases upon JEV infection. **(E)** Graph bar showing increased NF-κB luciferase activity at 6 and 12 h after JEV infection which later decreased 24 h post JEV infection. One-way ANOVA was used to determine statistical significance. *P* values were considered significant where *denotes *P* < 0.05, **denotes *P* < 0.005, and ***denotes *P* < 0.001 from 6 h sample, #P from 12 h sample. NF-κB luciferase vector was co-transfected with β-galactosidase vector. β-galactosidase activity was used to normalize luciferase activity. All experiments were repeated thrice and are represented as mean ± SE.

As JEV infection induced the expression of miR-146a in JaOArS982 strain, therefore we focused on this strain for our downstream studies. The levels of miR-146a target genes were checked after 24 h post JEV infection. The downregulation in TRAF6, IRAK1, and IRAK2 levels was observed 24 h post JEV infection as compared to uninfected cells used as control (Figure 2A). Since these genes are major adaptor molecules involved in NF-κB activation, the levels of phospho-p65, which is the subunit of NF-κB activator complex, were determined by Western blotting and found a decrease in ratio of phospho-p65 to total p65 post infection (Figure 2C). NF-κB luciferase construct containing NF-κB response element upstream firefly luciferase gene was used to ascertain the effect of JEV infection on NF-κB activation at various time points. β-galactosidase vector was co-transfected in order to normalize luciferase activity with β-galactosidase activity. NF-κB luciferase assay showed an increase in the NF-κB luciferase activity at 6 and 12 h post JEV infection, which later decreased at 24 h post infection. This confirmed the presence of a negative feedback loop, which suppresses the NF-κB activity after 24 h of JEV infection as compared to uninfected cells (Figure 2E). This depicted that miR-146a is suppressing NF-κB activation to create an anti-inflammatory milieu in the cell to facilitate its survival in host cells.

### miR-146a targets TRAF6, IRAK1, and IRAK2 genes

**Figure 3:**
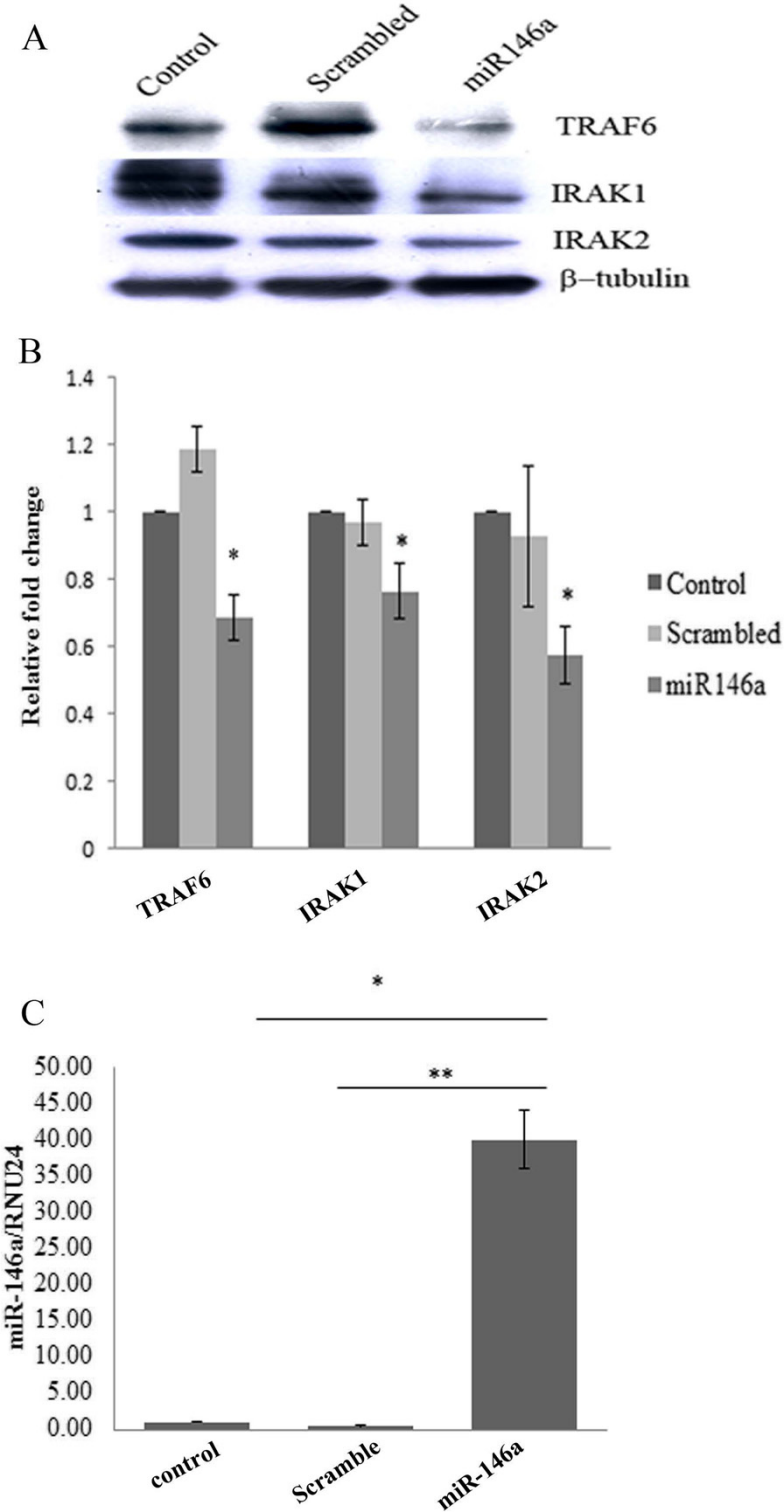
**miR-146a targets TRAF6, IRAK1, and IRAK2.** CHME3 cells were transfected with 100 pmol miR-146a mimic sequence. Scrambled seed sequence was used as control along with mock treatment as control. The cells were harvested 48 h post transfection. **(A)** Western blots showing downregulation of TRAF6, IRAK1, and IRAK2 genes upon miR-146a overexpression. **(B)** Densitometry plot showing downregulation of TRAF6, IRAK1, and IRAK2 genes upon miR-146a overexpression. The overexpressed samples were compared to scramble control for statistical analysis. **(C)** RT-PCR analysis of miR-146a levels in miR-146a overexpressed cells. The cells were harvested 48 h post transfection. miR-146a levels were determined by RT-PCR by using Taqman probe. RNU24 was used for normalization. All experiments were repeated thrice and are represented as mean ± SE. The fold change is significant where *denotes *P* < 0.05, **denotes *P* < 0.005, and ***denotes *P* < 0.001.

miR-146a targets genes involved in NF-κB activation. To confirm the targeting of TRAF6, IRAK1, and IRAK2 genes by miR-146a, miR-146a mimic was overexpressed in CHME3 cells. CHME3 cells were transfected by using 100 pmol of miR-146a and incubated for 48 h post transfection. Additionally, CHME3 cells were also transfected with scrambled miR-146a and used as a control. Significant upregulation of miR-146a was confirmed by TaqMan qPCR microRNA assay. TaqMan qPCR did not show any upregulation of miR-146a in CHME3 cells transfected with scrambled miR-146a (Figure 3C). The Western blot analysis has shown downregulation of TRAF6, IRAK1, and IRAK2 genes in the CHME3 cells overexpressing miR-146a (Figure 3A).

### Anti-miR-146a rescues TRAF6, IRAK1, and IRAK2 genes during JEV infection

**Figure 4:**
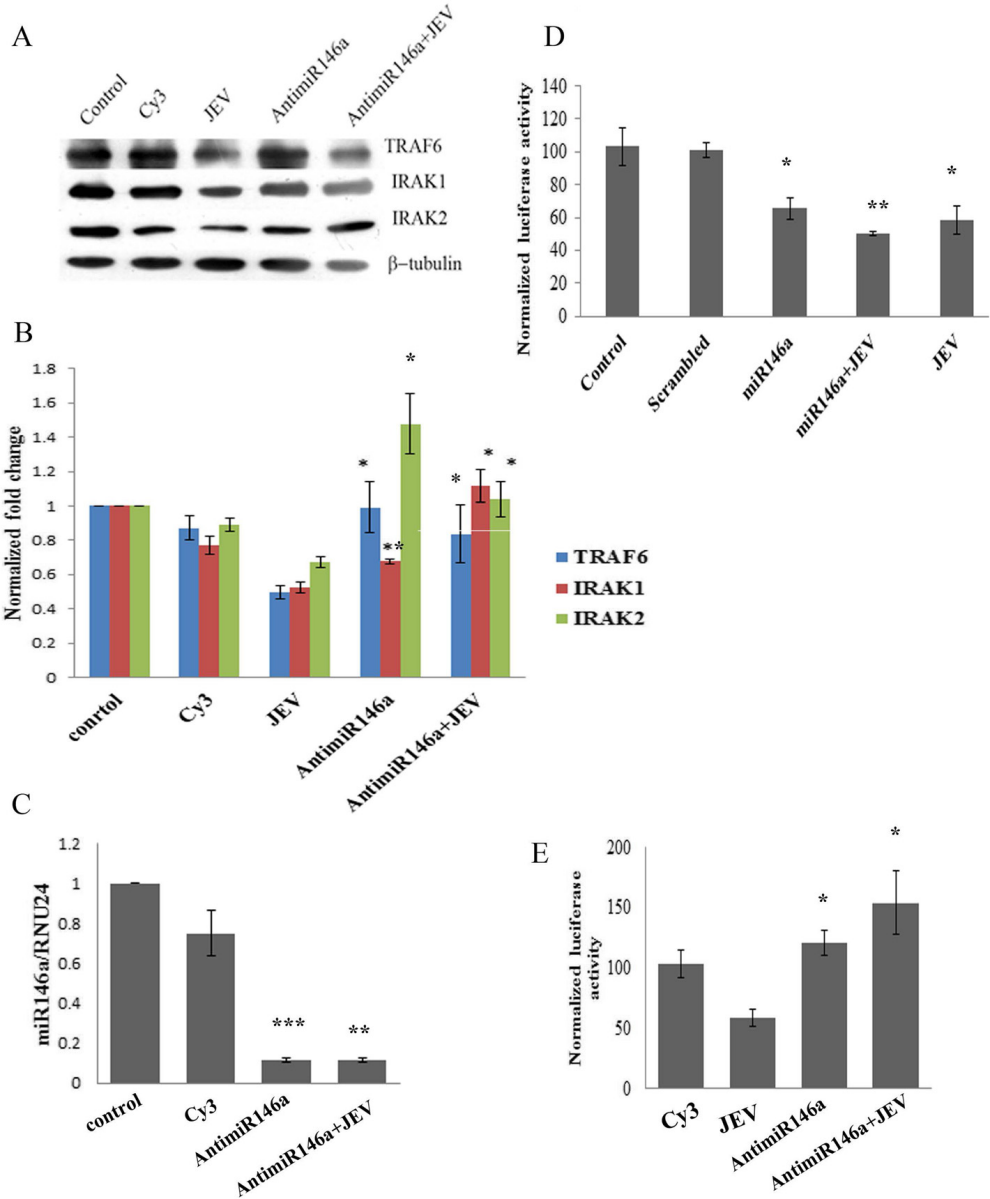
**Anti-miR-146a rescues TRAF6, IRAK1, IRAK2 genes.** CHME3 cells were transfected with 100 pmol anti-miR-146a. Cy3-labeled negative control and mock transfected cells were used as control. After 24 h of transfection, JEV infection was given (MOI 5). For better comparison, we also used a JEV-infected group untransfected with miR-146a mimic harvested after 24 h post infection. **(A)** Western blots showing upregulation of TRAF6, IRAK1, and IRAK2 genes upon silencing miR-146a. Anti-miR-146a also upregulated TRAF6, IRAK1, and IRAK2 genes after JEV infection. **(B)** Densitometry plot showing upregulation of TRAF6, IRAK1, and IRAK2 genes by anti-miR-146a w.r.t. JEV infection. The image density was normalized by β-tubulin. For statistical analysis, the protein levels of JEV-infected samples were compared to anti-miR-146a and anti-miR-146a + JEV samples. The fold change is significant where *denotes *P* < 0.05, **denotes *P* < 0.005, ***denotes *P* < 0.001. **(C)** Taqman RT-PCR analysis of anti-miR-146a-transfected samples to confirm silencing of miR-146a. No silencing was observed in Cy3-labeled negative control. Cy3-labeled control was used as control for *t*-test. **(D)** Graph bars showing reduced luciferase activity upon miR-146a overexpression. JEV infection after 24 h post miR-146a mimic transfection further reduced NF-κB activity. β-galactosidase activity was used to normalize luciferase activity. miR-146a was overexpressed, JEV was infected, and miR-146a + JEV samples were compared to scrambled control for statistical analysis. **(E)** Graph showing increased luciferase activity of NF-κB luciferase vector upon silencing of miR-146a by anti-miR-146a. Anti-miR-146a treatment 24 h prior to JEV infection also increased NF-κB luciferase activity. JEV-infected group was used for comparison for statistical analysis. All experiments were repeated thrice and are represented as mean ± SE. The fold change is significant where *denotes *P* < 0.05, **denotes *P* < 0.005, and ***denotes *P* < 0.001.

To further validate the role of miR-146a during JEV infection, we silenced miR-146a by using anti-miR-146a. Cy3-labeled scramble anti-miR was used as negative control. We confirmed the suppression of miR-146a by qPCR (Figure 4C). By Western blot analysis, we found that anti-miR-146a was able to rescue the expression of TRAF6, IRAK1, and IRAK2 genes from downregulation after JEV infection (Figure 4A). Increased expression of miR-146a led to the downregulation of TRAF6, IRAK1, and IRAK 2 genes, and anti-miR-146a was able to neutralize the effect of JEV on TRAF6, IRAK1, and IRAK2 genes.

### miR-146a suppresses NF-κB activation and cytokine production

To unveil the downstream effects of miR-146a overexpression which promoted JEV replication, the effect of miR-146a overexpression on NF-κB activation was analyzed. The NF-κB luciferase activity was found to be diminished by 40% in the presence of miR-146a, compared to scramble control (Figure 4D). JEV infection resulted into the downregulation of the luciferase activity by 50% in presence of miR-146a. To further delineate the suppressive effect of miR-146a, miR-146a activity was silenced by anti-miR-146a, which led to the increased luciferase activity. Elevated luciferase activity was observed in the presence of JEV (24 h post-infection) after anti-miR-146a transfection (Figure 4E). JEV infection also elevated NF-κB activity in the presence of anti-miR-146a, compared to untransfected JEV-infected cells (Figure 4E).

The effect of miR-146a overexpression was checked on cytokine production after JEV infection. JEV is known to trigger cytokine secretion in microglial cells. To see the effect of exogenous expression of miR-146a on JEV-induced cytokine production, the transcript levels of IL-6 were checked. We found reduced messenger RNA (mRNA) levels of IL-6 in miR-146a overexpressing cells, compared to scrambled control (Additional file 2: Figure S2A). The levels of TNF-α was also checked by ELISA of supernatants of JEV-infected miR-146a overexpressing CHME3 cells, and we found reduced TNF-α secretion (Additional file 2: Figure S2C). We also analyzed the IFN-β promoter activity in miR-146a overexpressing CHME3 cells after JEV infection and found 50% decrease in IFN-β promoter activity in the presence of miR-146a (Additional file 2: Figure S2E).

### miR-146a targets STAT1 gene

**Figure 5:**
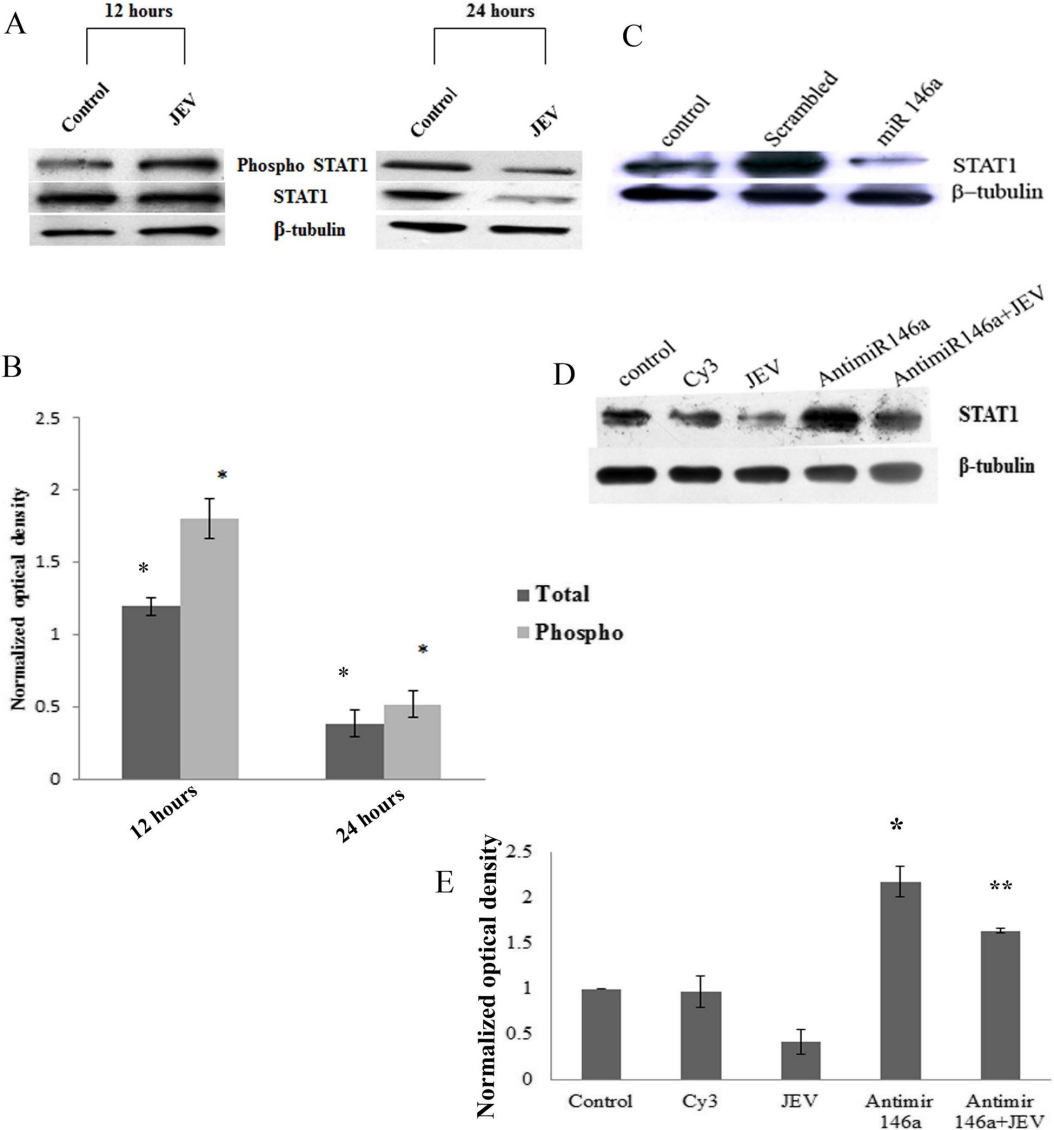
**miR-146a upregulation by JEV targets STAT1 phosphorylation. (A)** CHME3 cells were infected with JEV and pelleted at 12 and 24 h post infection. Western blots representing phospho-STAT1 and STAT1 levels at 12 and 24 h post infection. Both STAT1 and phospho-STAT1 got downregulated 24 h post infection. **(B)** Densitometry plot showing increase in phosphorylation of STAT1 at 12 h post infection. Later, no increase in phosphorylation was observed 24 h after infection due to downregulation in levels of STAT1. Both phospho-STAT1 and STAT1 image density was normalized by β-tubulin. For statistical analysis, the total and phospho-STAT1 levels of 12 and 24 h JEV-infected samples were compared to control uninfected samples at 12 and 24 h. **(C)** Western blot showing downregulation of STAT1 upon miR-146a mimic overexpression. **(D)** Western blot showing that silencing of miR-146a by anti-miR-146a upregulates STAT1 upon JEV infection. **(E)** Densitometry plot showing upregulation of STAT1 upon silencing of miR-146a. JEV infection also upregulates STAT1 when anti-miR-146a is transfected 24 h prior to JEV infection. The image density was normalized by β-tubulin. For statistical analysis, the anti-miR-146a and anti-miR-146a + JEV groups were compared to JEV-infected group. All experiments were repeated thrice and are represented as mean ± SE. The fold change is significant where *denotes *P* < 0.05, **denotes *P* < 0.005, and ***denotes *P* < 0.001.

miR-146a overexpression resulted in decreased IFN-β promoter activity, so the effect of miR-146a upregulation on interferon signaling was also investigated. The expression of STAT1 protein in miR-146a overexpressing cells was determined. Signal transducer and activator of transcription (STAT1) is a well-known downstream molecule in IFN signaling which binds to Janus kinases (Jak) and triggers expression of interferon-stimulated gene and is a known to target of miR-146a. The targeting of STAT1 gene by miR-146a was confirmed in our cells, and downregulation in STAT1 levels was observed upon miR-146a overexpression (Figure 5C). Anti-miR-146a rescued STAT1 from downregulation (Figure 5D).

Type I interferon activates STAT1 by its phosphorylation and dimerization. JEV has been reported to increase the STAT1 phosphorylation [30]. We observed upregulation in STAT1 phosphorylation (ser727) 12 h post JEV infection (Figure 5A). However, the level of total STAT1 was not changed in 12 h samples. Later, downregulation in total and phospho-STAT1 levels was noticed at 24 h post JEV infection (Figure 5A). We assume that downregulation in STAT1 levels would be due to miR-146a upregulation, which targets expression of STAT1. To confirm our hypothesis, miR-146a was silenced by using anti-miR-146a and found increased STAT1 levels in the presence of JEV (Figure 5D). So we speculate that miR-146a downregulates STAT1 levels which could hinder interferon signaling pathway.

### miR-146a suppresses JEV-induced ISRE promoter activity

**Figure 6:**
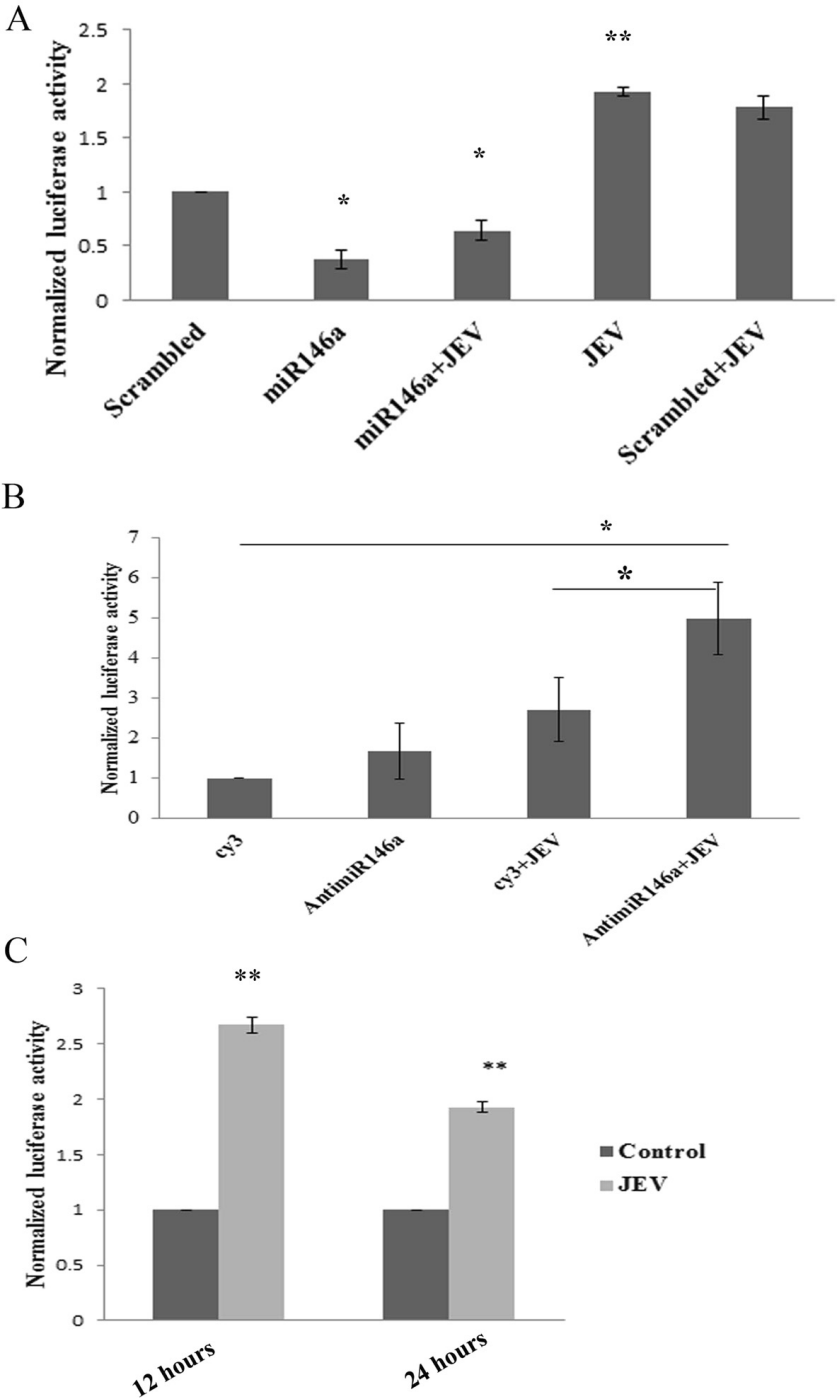
**miR-146a suppresses ISRE promoter activity.** CHME3 cells were co-transfected with 1 μg ISRE luciferase vector and 700 ng β-galactosidase vector to measure ISRE luciferase activity. **(A)** Graph bars showing reduced ISRE luciferase activity upon JEV infection in miR-146a overexpressing cells. miR-146a mimic was transfected along with vectors, and scrambled sequence was used as control for statistical analysis. Scrambled + JEV group did not show any significant decrease in ISRE activity upon JEV infection. JEV infection was given 24 h post transfection, and luciferase activity was measured after 24 h. **(B)** Graph bars showing increased ISRE luciferase activity upon JEV infection in anti-miR-146a transfected cells. Cy3-labeled scrambled anti-miR was used as control. Anti-miR-146a increased ISRE activity upon JEV infection as compared to scrambled Cy3 + JEV group. **(C)** Graph bars showing ISRE luciferase activity upon JEV infection at two time points - 12 and 24 h post infection. The ISRE activity decreases at later time point after JEV infection. All experiments were repeated thrice and are represented as mean ± SE. The fold change is significant where *denotes *P* < 0.05, **denotes *P* < 0.005, and ***denotes *P* < 0.001.

Activated STAT1 dimerizes and binds to other factors to enter the nucleus and activates the expression of interferon-stimulated genes after binding to interferon-stimulated response elements (ISRE) in the promoter of interferon-inducible genes. Since miR-146a reduced the STAT1 activation, the effect of miR-146a overexpression was further studied on ISRE activity by ISRE luciferase assay. miR-146a overexpressing cells displayed reduced luciferase activity, which depicted suppression of ISRE promoter activity upon JEV infection in miR-146a overexpressing cells (Figure 6A). However, we did not observe any significant decrease in ISRE activity in scramble transfected cells infected by JEV (Figure 6A). ISRE activity was increased upon JEV infection when miR-146a was silenced by using anti-miR-146a prior to JEV infection, compared to Cy3-labeled, scrambled transfected cells infected by JEV (Figure 6B).

To study the time-dependent effect of JEV infection on ISRE activity, the luciferase activity was checked at 12 and 24 h post JEV infection. ISRE activity got increased at early time point of 12 h of infection; but later, it decreased at 24 h post JEV infection (Figure 6C). JEV infection suppressed the ISRE activity after 24 h of JEV infection, which weakens the cellular immune response against the virus.

### Downregulation of interferon-stimulated genes by miR-146a

**Figure 7:**
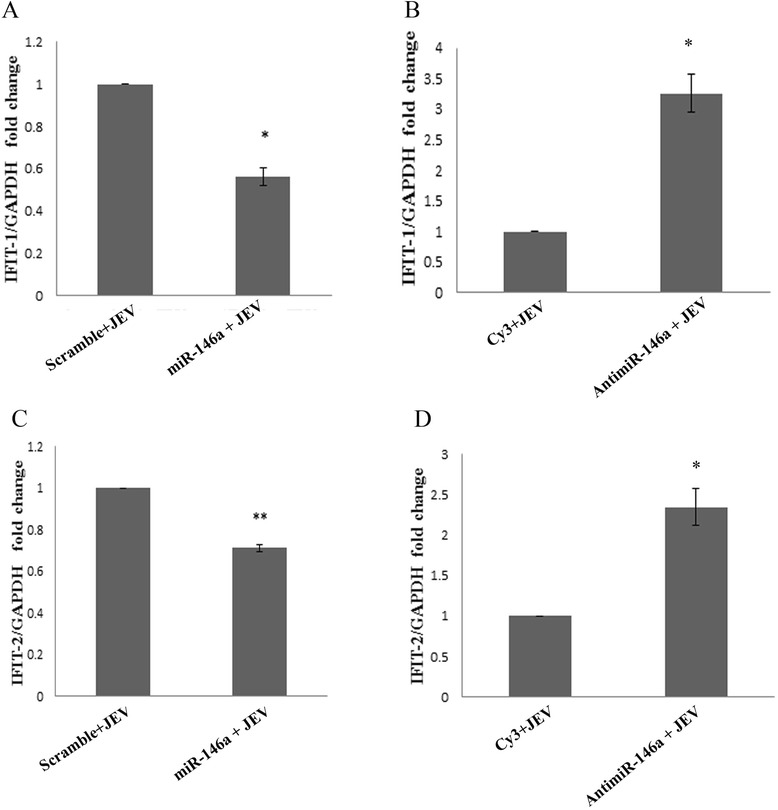
**miR-146a suppresses interferon-stimulated genes.** Effect of miR-146a overexpression on JEV-induced expression of interferon-stimulated genes. The levels of IFIT-1 and IFIT-2 were determined by RT-PCR. The C_T_ values were normalized by GAPDH levels, and fold change was calculated by 2^−∆∆C^_T_ method. **(A,C)** Graph bars showing decreased expression of JEV-induced IFIT-1 **(A)** and IFIT-2 **(C)** upon miR-146a overexpression as compared to scramble control. **(B,D)** Graph bars showing augmented IFIT-1 **(B)** and IFIT-2 **(D)** transcript levels upon silencing of miR-146a by anti-miR-146a. Cy3-labeled scramble sequence was used as negative control. All experiments were repeated thrice and are represented as mean ± SE. The fold change is significant where *denotes *P* < 0.05, **denotes *P* < 0.005, and ***denotes *P* < 0.001.

STAT1 acts as a transcription factor which increases the expression of many interferon-stimulated genes. Abrogation of STAT1 gene leads to reduced ISRE activity, which in turn affects the expression interferon-stimulated genes (ISGs). As we observed, miR-146a downregulated STAT1 levels; therefore, the levels of ISGs were determined after miR-146a overexpression. Interferon-induced protein with tetratricopeptide repeat (IFIT) proteins are major interferon-induced proteins. We checked the mRNA levels of two IFIT genes IFIT-1 and IFIT-2 after miR-146a overexpression. About 40% reduction in IFIT-1 mRNA levels (Figure 7A) and 30% reduction in IFIT-2 levels (Figure 7C) were noticed in cells, transfected miR-146a followed by JEV infection, compared to scramble control mimic. Anti-miR-146a reversed the effect of miR-146a by increasing the levels of IFIT-1 and IFIT-2 (Figure 7B,D). The miR-146a downregulates ISGs and disrupts Jak-STAT signaling.

### JEV modulates pro-inflammatory cytokines and expression of IFIT-1 and IFIT-2

**Figure 8:**
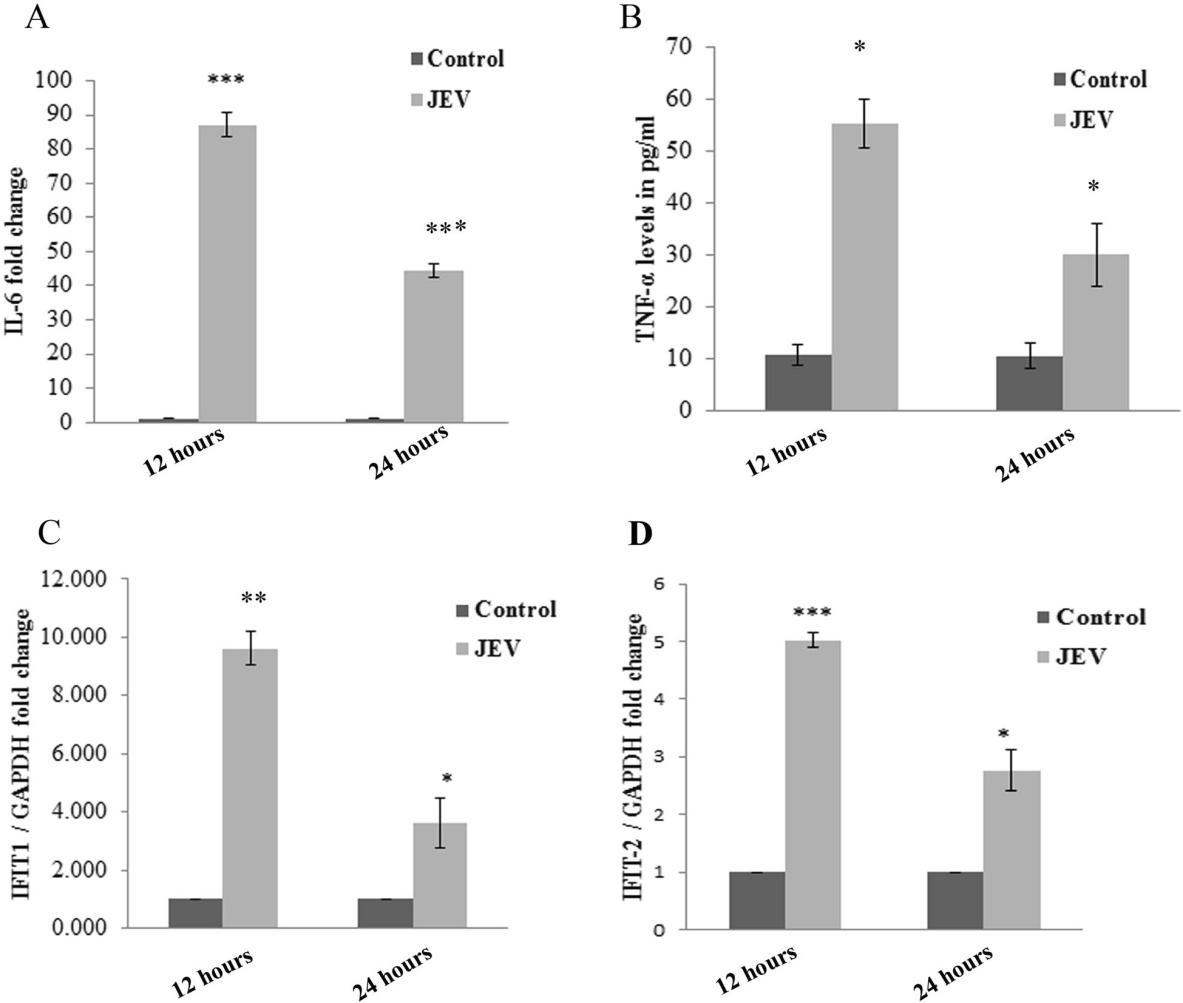
**JEV modulates pro-inflammatory cytokine expression and expression of IFIT-1 and IFIT-2.** CHME3 cells were infected by JEV, and the effect on expression of pro-inflammatory cytokines and interferon-induced genes (IFIT-1 and IFIT-2) was determined at 12 and 24 h post infection. **(A)** Graph showing the effect of JEV infection on expression of IL-6 at 12 and 24 h post infection. mRNA levels of IL-6 was determined by RT-PCR. Decrease in IL-6 levels was demonstrated at later time point of 24 h. **(B)** Graph plot showing increase in TNF-α secretion 12 h post JEV infection by ELISA. The TNF-α level was seen to be decreased after 24 h post infection. **(C,D)** Graph showing RT-PCR analysis of IFIT-1 **(C)** and IFIT-2 **(D)** transcript levels at 12 and 24 h after JEV infection. Increase in IFIT-1 mRNA level was found in early 12 h of infection but later decrease was observed (24 h). All experiments were repeated thrice and are represented as mean ± SE. The fold change is significant where *denotes *P* < 0.05, **denotes *P* < 0.005, and ***denotes *P* < 0.001.

Viruses elicit the secretion of pro-inflammatory cytokines in cells. This strategy is used by the host to restrict viral replication and survival. JEV triggers the innate immune response of the cells and elicits the secretion of pro-inflammatory cytokines like IL-6 and TNF-α. Significant upregulation in IL-6 and TNF-α levels were observed after JEV infection at early time point of 12 h. The decrease in levels of IL-6 and TNF-α was observed at late time point of 24 h (Figure 8A,B). To visualize the expression of interferon-stimulated genes after JEV infection in CHME3 cells, the cells were infected and the levels of IFIT-1 and IFIT-2 mRNA at 12 and 24 h post infection were determined. A significant increase was found in IFIT mRNA levels 12 h post infection, which decreased later at 24 h post infection (Figure 8C,D). So we infer that JEV downregulates IFIT levels and expression of pro-inflammatory cytokines at late hours of infection to suppress the cellular innate immune response to alleviate its survival in the cell.

## Discussion

JEV infection leads to neuroinflammation in JEV-infected patients, which leads to high morbidity and mortality. However, the survivors still show neurological and cognitive impairments [31]. JEV has been reported to evade the innate response of host and establish pathogenesis [32]. Recently, Hayasaka *et al.* reported that JEV JaOArS982 strain infection formed two different disease severity groups in mice, one of which succumbed mild infection with lower levels of TNF-α and IL-10 [33]. They got different results by using different JEV strains. They reported lower titer of JaOArS982 strain in the brain of infected mice, compared to the higher titer of other infective JEV strains, which elicited lower cytokine secretion in mice brain. This data is in support of our findings as our results are also strain specific, and we got decreased levels of miR-146a, in cells infected by JEV (P20778). This suggests that JEV JaOArS982 strain successfully shuts down the cellular inflammatory response to facilitate its survival. Interferon-induced Jak-STAT pathway activates anti-viral machinery of cells, which modulates cellular inflammatory response [34]. Viruses are known to target STAT1 to escape the IFN-induced anti-viral immune response. Simian [35] and mumps virus [36,37] abrogate Jak-STAT signaling by targeting STAT1. JEV has also been reported to abrogate the Jak-STAT pathway by blocking STAT1 phosphorylation and neutralizing IFN-α-induced anti-viral response [38]. Viruses modulate the cellular microRNA expression for their benefit [14]. miR-122 [39] has been reported to facilitate HCV replication. Vesicular stomatitis, dengue, and Chikungunya virus upregulate miR-146a to suppress cellular inflammatory response [19,20,21]. Enterovirus has been reported to induce miR-141 expression to suppress host translational machinery [40]. JEV has been reported to upregulate miR-29b to suppress the microglial activation [17] and miR-155 to suppress JEV-induced inflammatory response [18]. In this study, we deciphered the role of microRNA-146a in modulating innate immune response and JEV pathogenesis in human microglial cells.

We report the JEV-mediated increased expression of miR-146a in CHME3 cells. Our results are contradictory to findings of Pareek *et al.* who found downregulated miR-146a levels upon JEV infection [27]. This discrepancy may be due to different strains of virus used by Pareek *et al.* To confirm this strain-specific effect of JEV, we determined miR-146a levels in P20778 strain used by Pareek *et al.* and found similar results to their findings. This suggested that increased expression of miR-146a is strain specific. JEV infection downregulated the adaptor molecules TRAF6, IRAK1, and IRAK2 involved in NF-κB activation, which are targeted by miR-146a. To describe the specific role of miR-146a in targeting of these adaptor molecules, miR-146a was silenced and we found that anti-miR-146a rescued TRAF6, IRAK1, and IRAK2 from downregulation upon JEV infection. To further unveil the downstream effects on NF-κB activity, NF-κB promoter luciferase assay and Western blot analysis were performed. We found reduced luciferase activity at 24 h post JEV infection and reduced phosphorylation of NF-κB p65 subunit upon JEV infection. To confirm the presence of negative feedback loop, we checked the NF-κB luciferase activity at early time points (6 and 12 h) and found initial upregulation in luciferase activity followed by a decrease in luciferase activity at later hours. Initial NF-κB activation by JEV preludes the miR-146a overexpression, which further led to the downregulation of adaptor proteins involved in NF-κB activation and constitute a negative regulatory loop [41]. Hence, we conclude that JEV-mediated upregulation of miR-146a takes place to downregulate NF-κB activation.

miR-146a overexpression suppressed NF-κB activation as demonstrated by luciferase assay, and inhibition of miR-146a enhanced NF-κB activation upon JEV infection. Recently, Jin Ho Paik *et al.* also reported similar results upon miR-146a overexpression [42]. Our findings demonstrated that miR-146a creates an anti-inflammatory environment in cells. NF-κB subunits act as transcription factor for expression of pro-inflammatory cytokines. miR-146a overexpression also suppressed JEV-induced cytokine production (IL-6, TNF-α, IFN-β). The replication of JEV is increased upon miR-146a overexpression due to an anti-inflammatory environment created by overexpression of miR-146a. However, this enhanced replication of viral RNA was observed only at 24 h post infection and this effect reduced at later time points. To rule out the possibility that this reduced effect on viral replication could be due to degradation of overexpressed miR-146a at later hours, we checked the levels of miR-146a at later hours by RT-PCR and found that the level of miR-146a was retained in CHME3 cells (data not shown). We also found elevated levels of viral NS1 protein in miR-146a overexpressing cells. This may be due to greater accumulation of viral proteins in miR-146a overexpressing cells. Recently, Bing-Ching Ho *et al.* also reported that miR-146a supports the survival of *Enterovirus* in mice and silencing of miR-146a improved the survival of *Enterovirus*-infected mouse due to restoration of interferon production [43]. This suggested that the cellular anti-viral machinery got compromised in miR-146a overexpressing cells and supported viral replication. However, a recent study by Kundu *et al.* reported increased levels of SOCS1 and SOCS3 during early time points of infection which promoted JEV replication at early time points, but the viral titer got decreased due to the decrease in levels of SOCS1 and SOCS3 during later time points, which led to the activated cellular immune response [30]. We also found a decrease in viral mRNA levels at later time points. This decrease in viral titer in later time points may help the virus to persist latently in cells. Expression of a truncated form of viral NS1 protein and production of low virus titer in persistently JEV-infected murine neuroblastoma cells as an aftermath of virus-cell interaction [44] also depict that restriction of viral replication can assist viral persistence. Decrease in viral titer at later time points may be due to the activation of cellular immune machinery which nullified the effect of miR-146a overexpression.

miR-146a targets STAT1 gene which leads to abrogation of Jak-STAT pathway [45]. We also found similar results, where miR-146a overexpression reduced the STAT1 levels and inhibition of miR-146a upregulated the STAT1 levels. We also analyzed the effect of miR-146a overexpression on Jak-STAT pathway. Suppression of ISRE promoter activity and downregulated expression of interferon-stimulated genes (ISGs) were found in miR-146a overexpressing cells upon JEV infection. Interferon-induced protein with tetratricopeptide repeat (IFIT) proteins are well-known interferon-induced anti-viral proteins, having anti-proliferative effects [46]. IFIT-1 has been reported to restrict JEV replication [47]. IFIT-2 has been also reported to restrict VSV replication [48]. miR-146a downregulated the JEV induced expression of IFIT-1 and IFIT-2. Downregulation of IFIT levels perturb the cellular anti-viral machinery, which helps in JEV replication in host cells. Tang *et al.* has reported downregulation of other ISGs (OAS1, Mx1, LY6E) upon miR-146a overexpression [22]. These findings indicated that miR-146a abrogated the Jak-STAT pathway and downregulated the expression of ISGs, which led to the suppression of innate immune response against the virus and augmented the viral replication in miR-146a overexpressing cells.

We also analyzed the effect of JEV infection on STAT1 activation at different time points. JEV induced the STAT1 activation at early time points but downregulated the STAT1 levels at later time points due to targeting of the STAT1 by miR-146a. Inhibition of miR-146a led to the increased STAT1 levels upon JEV infection. The ISRE promoter activity and expression of IFIT-1 and IFIT-2 also displayed similar trends. We observed initial upregulation of ISRE promoter activity and levels of IFIT1 and IFIT2 upon JEV infection, which supported the activation of cellular innate immune machinery against the virus. At 24-h time point, we found decreased levels of IFIT1 and IFIT2 and reduced ISRE promoter activity which indicated that the virus has successfully suppressed the cellular inflammatory response. We observed the decreased expression of IL-6 and TNF-α at 24 h as compared to 12 h post JEV infection. These findings suggest that JEV-mediated miR-146a upregulation led to the suppression of NF-κB activation and STAT1 degradation, caused the downregulation of ISGs at later time point (24 h). Downregulation of ISGs has been reported to facilitate persistence of virus in cells [49]. This is a strategy embraced by JEV to suppress the cellular inflammatory response in human microglial cells and evade innate immune response. However; this effect is time dependent and other strategies adopted by the cell to combat viral replication could play an important role. The host cell triggers various anti-viral signaling pathways to curtail viral replication. Further studies are required to find out other anti-viral strategies adopted by JEV to evade cellular immune response. This study demonstrated strain-specific effects of JEV, as different JEV strains may lead to the varying downstream effects in host cellular immune responses. Understanding the regulatory role of cellular microRNAs during JEV infection in microglial cells would be helpful in understanding the molecular mechanism of JEV neuropathogenesis.

## Additional files

Additional file 1: Figure S1. JEV P20778 strain downregulates miR-146a and effect of miR-146a on P20778 replication. CHME3 cells were infected by JEV P20778 Vellore strain (MOI-5), and cells were harvested after 24 and 48 h for RNA isolation and qPCR. **(A)** Graph showing downregulation in miR-146a levels 24 and 48 h post infection obtained by RT-PCR using miR-146a-specific Taqman probes. Uninfected control groups of the same time points were used for comparison. RNU24 levels were used for normalization. Fold change was determined by 2^−∆∆C^_T_ method. **(B)** RT-PCR graph showing upregulated viral RNA levels in miR-146a overexpressing cells. CHME3 cells were transfected with 100 pmol miR-146a mimic, and P20778 JEV infection was given after 24 h. The cells were harvested 24 and 48 h post infection. Scrambled + JEV group of the same time points was used as control for comparison. Viral RNA level was determined by RT-PCR using JEV NS3 specific primers. The fold change was normalized by GAPDH RNA levels. All experiments were repeated thrice and are represented as mean ± SE. The fold change is significant where *denotes *P* < 0.05, **denotes *P* < 0.005, and ***denotes *P* < 0.001. Additional file 2: Figure S2. miR-146a suppresses cytokine expression. Graph bars showing the effect of overexpression of miR-146a on various cytokines upon JEV infection. miR-146a suppressed the expression of JEV-triggered cytokines (IL-6, TNF-α, IFN-β). **(A,B)** RT-PCR analysis of IL-6 levels in JEV-infected miR-146a overexpressing cells. (A) miR-146a suppressed the IL-6 expression as compared to scramble control. Anti-miR-146a transfection increased the expression of IL-6 upon JEV infection as compared to Cy3-labeled negative control. **(B-D)** Graph bars showing ELISA of supernatants to check TNF-α secretion in miR-146a overexpressing cells (C) and in anti-miR-146a transfected cells (D) upon JEV infection. **(E-F)** Graph depicting the effect of miR-146a on IFN-β promoter activity by IFN-β promoter luciferase assay. miR-146a suppressed the JEV-triggered IFN-β promoter activity (E) whereas anti-miR-146a enhanced the IFN-β promoter activity upon JEV infection (F). The infected samples were compared to uninfected control for statistical analysis. All experiments were repeated thrice and are represented as mean ± SE. The fold change is significant where *denotes *P* < 0.05, **denotes *P* < 0.005, and ***denotes *P* < 0.001.
