# Supplementary figures and images for: miR-146a suppresses cellular immune response during Japanese encephalitis virus JaOArS982 strain infection in human microglial cells
Source: J Neuroinflammation. 2015 Feb 18;12:30. doi: 10.1186/s12974-015-0249-0 (PMC4355369; doi:10.1186/s12974-015-0249-0)

**Supplementary fig-1 : JEV P20778 Strain downregulates miR-146a and effect of miR-146a on P20778 replication.**

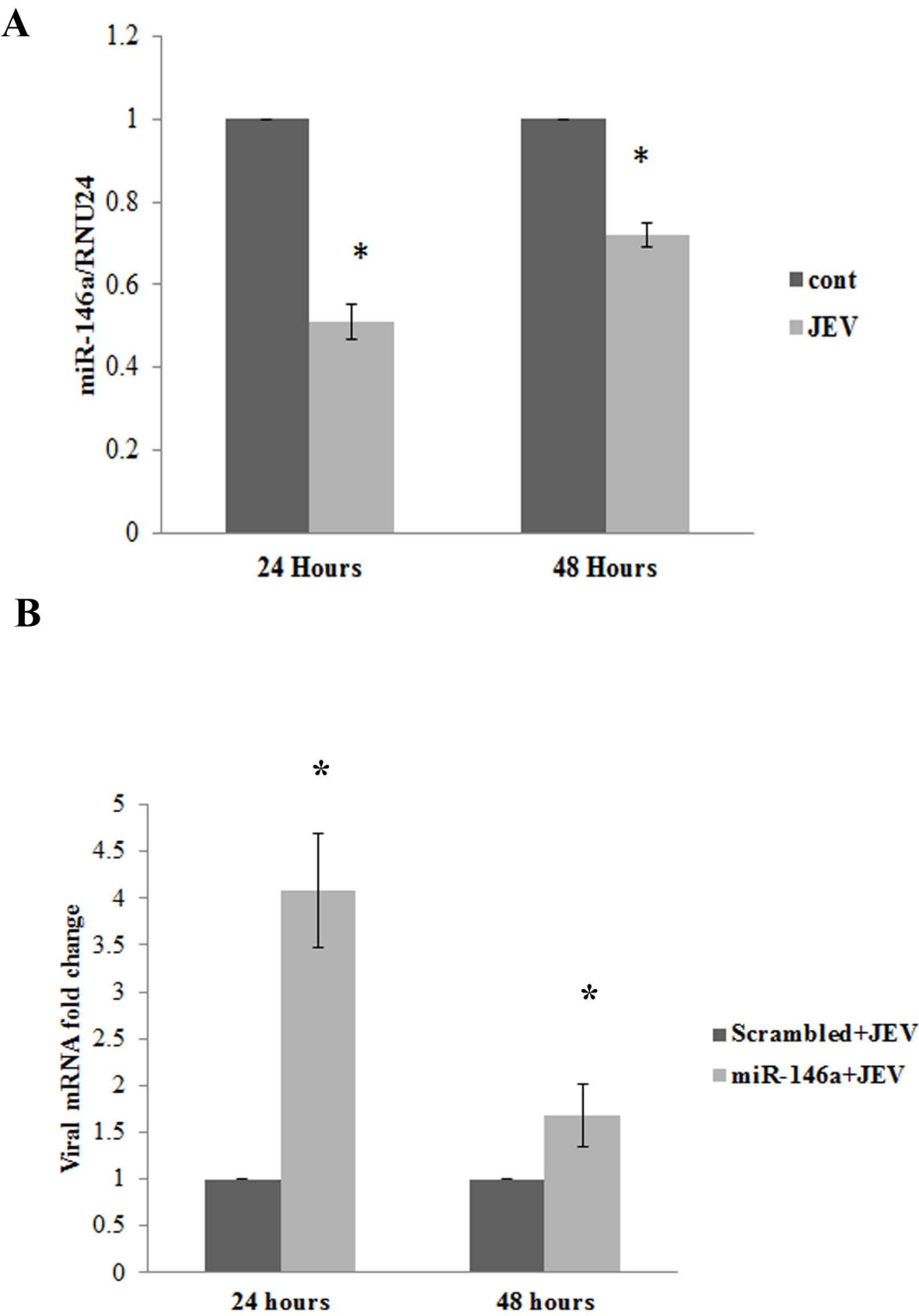

Supplement: Additional file 1: Figure S1. — JEV P20778 strain downregulates miR-146a and effect of miR-146a on P20778 replication. CHME3 cells were infected by JEV P20778 Vellore strain (MOI-5), and cells were harvested after 24 and 48 h for RNA isolation and qPCR. (A) Graph showing downregulation in miR-146a levels 24 and 48 h post infection obtained by RT-PCR using miR-146a-specific Taqman probes. Uninfected control groups of the same time points were used for comparison. RNU24 levels were used for normalization. Fold change was determined by 2−∆∆C T method. (B) RT-PCR graph showing upregulated viral RNA levels in miR-146a overexpressing cells. CHME3 cells were transfected with 100 pmol miR-146a mimic, and P20778 JEV infection was given after 24 h. The cells were harvested 24 and 48 h post infection. Scrambled + JEV group of the same time points was used as control for comparison. Viral RNA level was determined by RT-PCR using JEV NS3 specific primers. The fold change was normalized by GAPDH RNA levels. All experiments were repeated thrice and are represented as mean ± SE. The fold change is significant where *denotes P < 0.05, **denotes P < 0.005, and ***denotes P < 0.001. [file 12974_2015_249_MOESM1_ESM.pdf]

## Supplementary fig-2: miR-146a suppresses cytokine expression

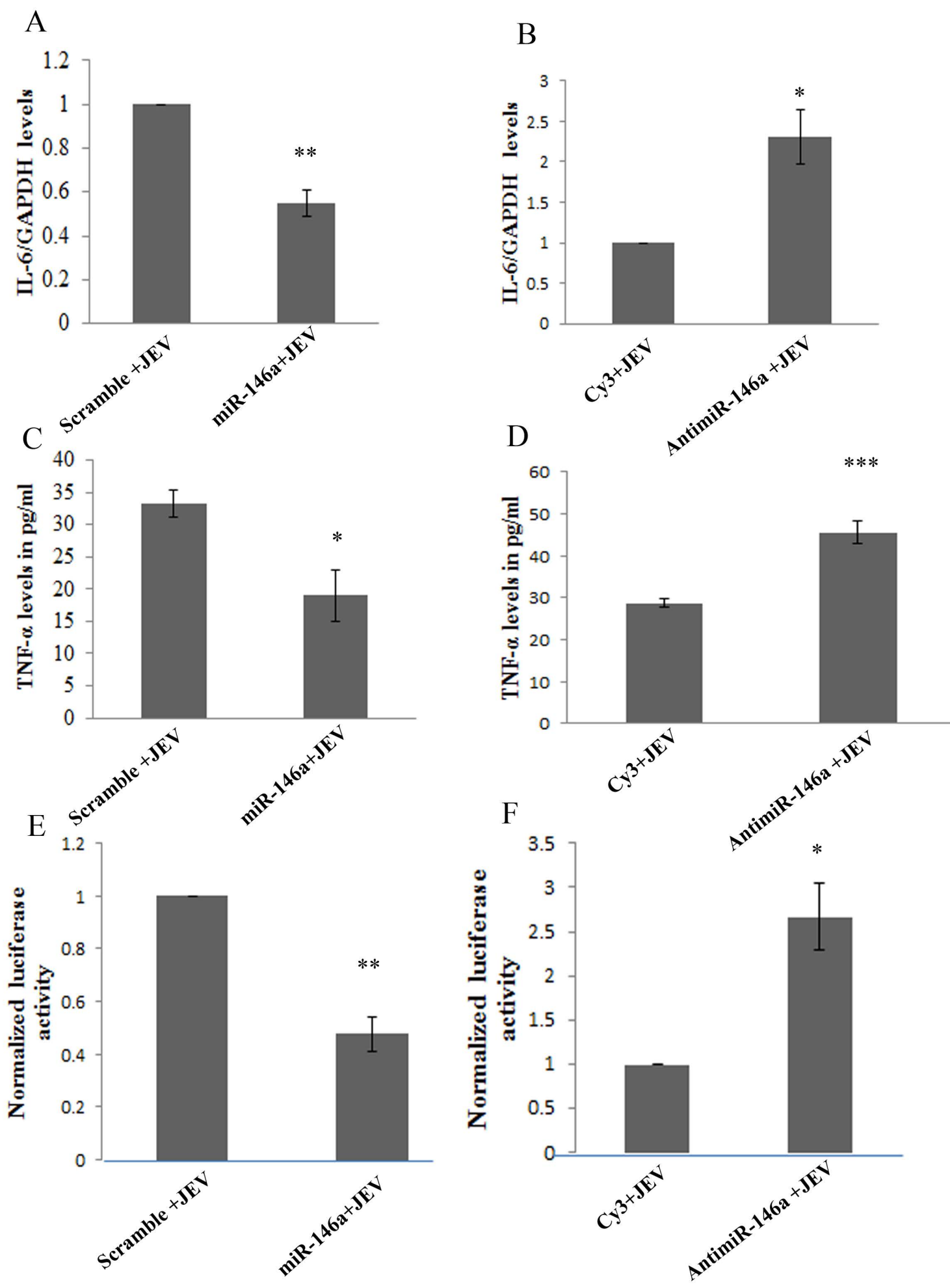

Supplement: Additional file 2: Figure S2. — miR-146a suppresses cytokine expression. Graph bars showing the effect of overexpression of miR-146a on various cytokines upon JEV infection. miR-146a suppressed the expression of JEV-triggered cytokines (IL-6, TNF-α, IFN-β). (A,B) RT-PCR analysis of IL-6 levels in JEV-infected miR-146a overexpressing cells. (A) miR-146a suppressed the IL-6 expression as compared to scramble control. Anti-miR-146a transfection increased the expression of IL-6 upon JEV infection as compared to Cy3-labeled negative control. (B-D) Graph bars showing ELISA of supernatants to check TNF-α secretion in miR-146a overexpressing cells (C) and in anti-miR-146a transfected cells (D) upon JEV infection. (E-F) Graph depicting the effect of miR-146a on IFN-β promoter activity by IFN-β promoter luciferase assay. miR-146a suppressed the JEV-triggered IFN-β promoter activity (E) whereas anti-miR-146a enhanced the IFN-β promoter activity upon JEV infection (F). The infected samples were compared to uninfected control for statistical analysis. All experiments were repeated thrice and are represented as mean ± SE. The fold change is significant where *denotes P < 0.05, **denotes P < 0.005, and ***denotes P < 0.001. [file 12974_2015_249_MOESM2_ESM.pdf]
